# Immigrant and ethnic minority patients` reported experiences in psychiatric care in Europe – a scoping review

**DOI:** 10.1186/s12913-023-10312-1

**Published:** 2023-11-21

**Authors:** Marte Karoline Råberg Kjøllesdal, Hilde Hestad Iversen, Kjersti Eeg Skudal, Lina Harvold Ellingsen-Dalskau

**Affiliations:** 1 Department of Public Health Science, Norwegian University of Life Sciences, Postboks 5003, 1433 Ås, Norway; 2 Center for Evidence-Based Public Health: A Joanna Briggs Institute Affiliated Group, Ås, Norway; 3 Norwegian Institute of Public Health, Health Services Research, Postboks 222 Skøyen, 0213 Oslo, Norway

**Keywords:** Psychiatric care, Immigrants, Patient experiences, Ethnic minority

## Abstract

**Background:**

There is little evidence on experiences in psychiatric care treatment among patients with immigrant or ethnic minority background. Knowledge about their experiences is crucial in the development of equal and high-quality services and is needed to validate instruments applied in national patient experience surveys in Norway. The aim of this scoping review is to assess and summarize current evidence on immigrant and ethnic minorities` experiences in psychiatric care treatment in Europe.

**Methods:**

Guidelines from the Joanna Briggs Institute were followed and the research process adhered to the Preferred Reporting Items for Systematic Reviews and Meta-Analyses extension for Scoping Reviews. The literature search was carried out in Medline, Cinahl, Web of Science, Cochrane database of systematic reviews, Embase, and APA PsychInfo, up to Dec 2022, for articles on immigrant patients` experiences in psychiatric care. Reference lists of included articles were screened for additional relevant articles. Titles and abstracts were screened, and potentially relevant articles read in full-text, by two researchers. Evidence was extracted using an a priori extraction form and summarized in tables and text. Any disagreement between the reviewers regarding inclusion of articles or extracted information details were resolved through discussion between authors.

**Results:**

We included eight studies in the scoping review. Immigrant and ethnic minority background patients did not differ from the general population in quantitative satisfaction questionnaires. However, qualitative studies showed that they experience a lack of understanding and respect of own culture and related needs, and difficulties in communication, which do not seem to be captured in questionnaire-based studies.

**Conclusion:**

Raising awareness about the importance of respect and understanding for patients` cultural background and communication needs for treatment satisfaction should be addressed in future quality improvement work.

**Supplementary Information:**

The online version contains supplementary material available at 10.1186/s12913-023-10312-1.

## Background

Mental health issues represent a substantial share of contacts with health care services in Europe and is a major public health issue [1]. It`s prevalence is not evenly distributed across the population, and international studies [2], as well as studies from Norway [3], Sweden [4] and Denmark [5] find that both self-reported levels and risk of diagnoses are higher in some groups of immigrants than in the general population. Despite the elevated prevalence of mental health disorders among immigrants, the use of psychiatric care services is lower than in the majority population in Norway [6, 7]. Reasons for differences in use of services by those with immigrant background may include practical barriers such as language, cultural barriers and stigma and low health literacy and knowledge of services, but also differences in quality of services and perceived benefit of treatment.

Patient experiences are an important aspect of quality of care and could inform service providers and policy makers of strengths, weaknesses and areas for improvement in the services [8]. Good patient experiences are related to improved communication, correct diagnoses and adherence to therapy and can promote service user participation in own care [8]. Studies assessing patient experiences with mental health care have measured experiences related to interpersonal relationships, respect and dignity, access and care coordination, drug therapy, information, psychological care and care environment [9, 10]. Most studies on experiences in mental health care are carried out among patients from a majority background [11]. However, needs and expectations and thus the experiences and satisfaction of patients are influenced by a range of factors, including knowledge about care, values and culture and sociodemographic factors [11]. Some studies indicate that patients with an immigrant background have lower satisfaction with these services than others [11]. Migrant populations may have expectations of care and explanatory models of mental health which differ from those of majority populations, traumatic life-events, migration-related stress, experiences of discrimination, cultural preferences and limited proficiency in host language influencing their experiences in health care. The knowledge on immigrant patients` experiences in mental health care is fragmented and scarce. Knowledge about which elements immigrants regard as most important for the treatment to be useful and good for them is therefore needed to develop services which are equal and of high quality for all.

In Norway, continuous, electronical measurements of patient perceived quality in mental health services have been carried out since 2020, as part of the national quality monitoring and improvement of health services [12]. In developing these measurements further, one important aspect is to focus on experiences among immigrants. Specifically, this means to assess and include areas of special importance for immigrant patients in measurement tools, and to implement means to increase the response rate among immigrant patients (e.g. reduce the length of the questionnaire, offer translated versions). In a preliminary literature search we found no existing review on immigrant patients` experiences in mental health care. Thus, our objective was to synthesize evidence on experiences immigrant patients have in psychiatric care in Europe and provide an overview of areas of particular importance to validate instruments applied in national patient experience surveys in Norway. Our research questions are: Which experiences do patients with immigrant and ethnic minority background have in psychiatric care? Do their experiences differ from those of other patients? A scoping review is an evidence synthesis that identify and map the breadth of available evidence on a particular field and identifying key factors related to a concept [13]. This makes scoping review a suitable approach to our research questions, which best can be answered by synthesizing evidence from a range of studies, including both quantitative and qualitative. Results from quantitative studies will provide knowledge regarding experiences captured by existing questionnaires on patient reported experiences, whereas qualitative studies will inform us about experiences important to immigrants which are not captured by currently used questionnaires, but which should be considered included in future quantitative surveys.

## Methods

The Joanna Briggs Institute`s methodology for scoping reviews was followed [14] and the research process reported according to the Preferred Reporting Items for Systematic Reviews and Meta-Analyses extension for Scoping Reviews [15] (Appendix 1). The protocol for the scoping review was published in Open Science Framework (http://osf.io/pce52/).

### Search strategy

Our point of departure for this scoping review was a need to develop and improve questionnaires used to capture patient experiences both in psychiatric care and substance abuse treatment. The literature search thus entailed both psychiatric care and substance abuse treatment. Only one study on patient experiences among immigrant patients in substance abuse treatment was found, and this was carried out among men with co-occurring substance use- and mental health disorders. Thus, the focus of the final scoping review was on patient experiences in psychiatric care only.

A previous literature search carried out for patient experiences in substance abuse treatment was used as a starting point for developing the search strategy [16], and search words and terms related to psychiatric care and to immigrant status or being an ethnic minority were included. We included search terms related to both patient experiences and patient satisfaction. Patient satisfaction relates to a patient`s subjective evaluation of the service in general. Patient experiences focus more on factual information about processes and events, and its relation to patient satisfaction is dependent on a person`s standards and expectations [17]. Both types of measures reflect health care quality, but patient experiences may be more informative for quality development work. The search strategy was developed by the authors in close collaboration with an experienced librarian at the Norwegian Institute of Public Health. The search strategy, including all identified keywords and index terms, was adapted for each included database (Full search strategy included in Appendix 2). The search was carried out in Medline, Cinahl, Web of Science, Cochrane database of systematic reviews, Embase, APA PsychInfo, up to Dec 2022. The final search was carried out 15.12.2022. A starting date was not applied for our search, and it was limited to articles in English or a Scandinavian language, based on language competencies of the research team. Reference lists of included articles, as well as related reviews identified in the search, were screened for additional relevant studies.

**Fig. 1:**
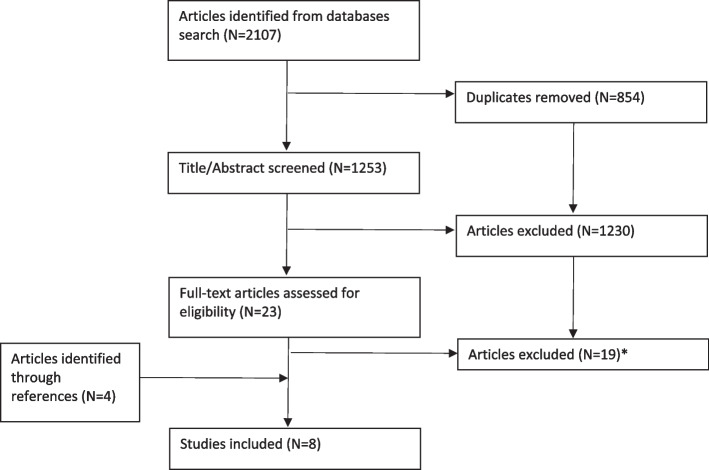
Flow diagram for included studies. *Reason for exclusion given in Appendix 3, Supplementary Table 1

All identified references were collated, uploaded into EndNote 20 and deduplicated. Titles and abstracts were screened by two of the authors (MKK, LED) for assessment against the inclusion criteria for the review. Potentially relevant articles were read in full text by the same two authors. Any disagreements between the reviewers at each stage of the selection process were resolved through discussion. The two authors agreed upon > 98% of the articles based on title/abstract screening and all the articles in the full text screening. The results of the search and the study inclusion process, including reasons for exclusion after full-text screening is reported in Fig. 1 and in Appendix 3, Supplementary Table 1.

The scoping review includes peer-reviewed articles reporting from quantitative and qualitative studies on patient experiences and satisfaction in psychiatric care in Europe, and with a focus on patients with immigrant or ethnic minority background. We included studies both from inpatient and outpatient care and both former and current patients. In the protocol, we focused on experiences of immigrants in inpatient care. But as the number of articles reporting on this was low (*N* = 3), we decided to widen our scope to include outpatient settings, and to also include ethnic minority patients with background from a country other than their country of residence (not indigenous people). Ethnic minorities may have lived in their country of residence for generations and do thus often not experience challenges related to being new in a country, such as language and lack of knowledge of the health systems. They may, however, face cultural differences in views of mental illness and treatment and discrimination based on their physical appearance or cultural traditions. A few references were focusing on differences by race in the US. As the sociocultural and historical context in Europe and the US is rather different, we have chosen to include only articles from Europe, and excluding those not from Europe. We included articles on patients 16 years or older, but not articles reporting experiences in children and adolescent mental health services. We excluded studies carried out in the general population and not focusing on specified service user groups.

All included articles were subjected to a data extraction procedure. We used an a priori extraction form, based on the JBI guidelines for scoping reviews [14] (Appendix 4), to systematically extract information from the studies. To ensure that all relevant information was extracted, two authors (MKK, LED) independently extracted information following the pre-defined categories; author, publication year, title, aim, country, context, participants, questionnaire applied (if relevant), domains of experiences assessed, results (main points). Any disagreement between the reviewers regarding extracted information details were resolved through authors discussing and agreeing upon what was of relevance for the scoping review. The extracted information was summarized in tables and a narrative synthesis.

## Results

A total of 1253 unique articles were identified through the database searches (Fig. 1). After screening of titles and abstracts, 23 articles were read in full-text and of those 4 were included in the scoping review. In addition, 4 eligible articles were identified through references screening and included, giving a total of 8 included studies. The included articles are presented with some selected descriptive details in Appendix 3, Supplementary Table 2.

### Description of included studies

Characteristics of the eight included articles in our scoping review are presented in Table 1. Of the eight studies, five were quantitative [18–22] and three qualitative [23–25]. Three studies reported from an in-patient setting [18, 19, 21], four from both in-patient and out-patient settings [22–25] and one from out-patient setting only [20]. Four studies were from the UK [18, 19, 23, 24], one from Denmark [20], one from Germany [22], one from Norway [25] and one reported from several countries [21]. The studies were published in the period 1999–2022, with more than half (5 articles) published from 2019 onwards. The studies from the UK reported on experiences among ethnic minorities, and the other studies among migrants. The quantitative studies included between 216 and 7302 participants, and four studies included patients both with and without a migrant/ethnic minority background [18, 19, 21, 22], whereas one study included only immigrants [20]. Two of the qualitative studies conducted individual interviews only [24, 25] and one did focus group sessions in addition to individual interviews [23]. The number of participants in the qualitative studies varied between 8 and 26, and all three studies included immigrants or ethnic minorities only [23–25]. The studies from UK focused on ethnic minorities [18, 19, 23], except one which focused on immigrants [24]. The other studies focused on immigrants [20–22, 25], and the term immigrant most often included both immigrants and their descendants (first- and second-generation immigrants).

**Table 1:** Characteristics of included studies

Study characteristics	Number of studies	References
*Study design*		
Quantitative	5	[18–22]
Qualitative	3	[23–25]
In-patient	3	[18, 19, 21]
Out-patient	1	[20]
Both in- and out-patient	4	[22–25]
*Country*		
UK	4	[18, 19, 23, 24]
Germany	1	[22]
Norway	1	[25]
Denmark	1	[20]
Several (Germany, Belgium, UK, Poland and Italy)	1	[21]

Among the quantitative studies, 4 used an existing questionnaire for measuring patient experiences and satisfaction [18, 19, 21, 22] and one study used a new questionnaire that was developed based on the literature, other questionnaires and clinical experience [20]. Two studies included a question about satisfaction with mental health treatment in general [18, 20], and four made a score of overall satisfaction based on questions on experiences and satisfaction in multiple domains of the treatment [18, 19, 21, 22] (Table 2). Three studies specified satisfaction with medication and with other aspects of treatment [19, 21, 22]. All studies included questions on experiences with staff and their professional and interpersonal skills. Three studies included questions regarding helpfulness of treatment and feeling better after treatment [19–21]. Two studies included questions related to the ward and its social and physical qualities [18, 19], and two included satisfaction with information given [20, 22]. Only one study [22] included questions on relatives’ involvement, and two on cultural aspects related to treatment [19, 20].

**Table 2:** Use of questionnaires and domains assessed in quantitative studies

	Number of studies	References
Used existing questionnaire for patient experiences	4	[18, 19, 21, 22]
Developed own questionnaire for patient experiences	1	[20]
Single question about general satisfaction	2	[18, 20]
Composite measure of general satisfaction	4	[18, 19, 21, 22]
*Included domains*		
Satisfaction with treatment	5	[18–22]
Staff (professional and interpersonal skills)	5	[18–22]
Medication	3	[19, 21, 22]
Treatment outcome/helpfulness of treatment	3	[19–21]
Ward (social and physical qualities)	2	[18, 19]
Satisfaction with information given	2	[20, 22]
Relatives involvement	1	[22]
Cultural aspects of treatment	2	[19, 20]

Among the qualitative studies, two assessed experiences with mental health services among patients with South Asian background in UK [23, 24], and one assessed experiences among patients with an immigrant background (first and second generation) in Norway [25].

### Synthesis of evidence

#### Comparison of satisfaction with services between patients with immigrant or ethnic minority background and majority background

Two studies compared satisfaction with in-patient mental health treatment among White British patients and ethnic minority patients in UK [18, 19]. One study found no ethnic differences in satisfaction with treatment, but that White patients reported more adverse events than others [18]. The other found no ethnic differences in most domains (16 of 21) of treatment, but that Black patients were less likely than White to perceive that they receive the right treatment or get the right medication [19]. Anderson et al. [21] and Gaigl et al. [22] compared experiences among mental health patients with and without a migration background, with diverging results. In a multisite study in both in- and out-patient settings across Germany, Italy, Poland, Belgium and UK, Anderson et al. found that migrants were less satisfied with their treatment than non-migrants. In an in-patient setting in Germany, patients with a migrant background had higher overall satisfaction with treatment and with involvement of relatives [22], and first-generation immigrants had higher satisfaction overall and with the professionals, efficacy and involvement of relatives compared to the second-generation immigrants and non-immigrants. There are no studies to assess differences in patient experiences in mental health care between various immigrant groups.

#### Domains of care highlighted as important by immigrants and ethnic minorities

##### Cultural understanding and communication

In all the three qualitative studies, the importance of professionals understanding and respecting their cultural background was highlighted [23–25]. Participants expressed that this was not in place when they had received treatment, and that this led to a lack of connection to the professionals and lower satisfaction with services. Some said that having a professional of same, or at least a minority, background, could have helped. Participants in the Norwegian study made a point that their needs were different from those of patients with the same diagnoses from the host population, and that the services were not tailored to meet their needs [25]. They experienced to receive a large extent of the treatment in group settings, with no adjustment to their command of the Norwegian language or understanding or to their individual or cultural starting point to work with their disorder. In one quantitative study assessing experiences among patients of a non-Western refugee background in a competence centre for transcultural psychiatry [20], the satisfaction with the treatment was overall high, including the cultural understanding. The special setting could have helped to a high score on experiences with the professionals cultural understanding. Nevertheless, a perception of being met with respect for own culture was related to higher overall satisfaction, highlighting its importance for patients with an immigrant background. This dimension of care was not assessed in the other quantitative studies.

Patients of ethnic minority background in two qualitative studies expressed frustration related to communication, gaining a deep understanding of what was said, challenges in expressing themselves in the host language and the lack of interpreters [23, 24]. Participants in one study also expressed a lack of information about diagnoses and treatment [24]. In the quantitative studies, three of them described that participants had to be able to communicate in the host language to participate [19, 21, 22], and one offered interpreters and translated questionnaires [20]. However, no questions on language and communication were included in questionnaires.

##### Relatives’ involvement and aftercare

In two of the qualitative studies, participants expressed that having relatives with them and supporting them was, or would have been helpful [24, 25]. Participants also mentioned stigma related to mental illness in their communities and that raising awareness of these disorders and reducing stigma would help them seek treatment and benefit from it [23, 25].

##### Practical issues and service development

Some participants in qualitative studies mentioned that they experienced practical barriers to practice religion when being in treatment, e.g., having a place to pray [23]. A concern was also raised that their voices were not heard in service development, which contributed to services less useful for them [23].

## Discussion

The limited number of quantitative studies comparing satisfaction scores between immigrant or ethnic minority patients and majority patients exhibit diverging methods and results, and do not provide evidence that immigrant or ethnic minority patients overall report poorer satisfaction with mental health treatment than the majority. However, evidence from qualitative studies suggests that immigrant patients experience a lack of understanding and respect of own culture and related needs, as well as difficulties in communication due to both cultural differences and language barriers. These issues were closely related to experiences of poorer satisfaction with mental health services. Importantly, these aspects were not captured in quantitative studies, except for one study from a competence centre for transcultural psychiatry [20].

Several of the aspects highlighted in the included studies, such as communication difficulties related to language and lack of insight, interest, and respect for the patient`s migration background by practitioners, have also been highlighted in studies conducted in general health care services [26]. Issues of discrimination and an expressed wish to be treated as a person have also been raised [26]. Thus, it seems that some of the areas of importance to immigrants in mental health care, also apply to other and more general parts of the health service system. Nevertheless, these are issues important to address in developing mental health care quality and in measuring perceived quality of this care. Overall satisfaction with primary health care services among immigrants vary across studies, like we have reported for mental health services in this scoping review. Some studies report lower satisfaction with primary health care services among immigrants than among others [27], whereas other studies report high satisfaction, including the perception of being met with respect for cultural background and own wishes [28, 29]. Due to the heterogeneity and the relatively low number of included studies in this scoping review, it is not possible to draw any conclusions on differences in satisfaction with mental health services between immigrants and ethnic minorities. Potential differences could be due to differences in language proficiency, knowledge of the health care system and to expectations to the mental health care services.

Satisfaction is related to expectation. For example have Vietnamese immigrants in the UK been shown to report better satisfaction, but also lower expectations, to health care than others [30]. Some studies have suggested the presence of a “happy migrant effect”, describing immigrants rating the quality of services as good because they compare it to bad experiences in their country of origin or during the migration process, or because of a feeling that they should have been thankful or have a poor self-esteem arising from language challenges [20]. This could also be a current issue in mental health care, as immigrants come from very different health systems in their country of origin, sometimes non-existent in terms of mental health services. Our results did not show a consistently higher satisfaction among patients with an immigrant background. Therefore, it is not possible to draw any conclusions regarding how expectations may be influencing perceptions of the quality of mental health care.

It is generally expected that good patient experiences are associated with improved outcomes, as they contribute to a higher involvement of patients in own care through better communication, correct diagnoses and adherence to therapy. However, the included studies do not provide a clear confirmation of this relationship. One of the studies included objective measures of treatment outcomes [21], while another study relied on self-report data [20]. Anderson et al. [21] found that despite immigrants reporting a slightly lower satisfaction with mental health services, there were no differences between immigrants and others in objective outcomes including length of stay, rehospitalizations or number of untoward events. In fact, immigrants even had a lower rate of suicide than non-immigrants. In a competence center for transcultural psychiatry, patients who experienced a subjective improvement also reported better satisfaction scores [20].

### Strengths and limitations

The strengths of this study were a thorough search in six databases covering medicine, mental health literature and social science. The authors have followed guidelines from the Joanna Briggs Institute and adhered to the Preferred Reporting Items for Systematic Reviews and Meta-Analyses extension for Scoping Review. We chose to include studies from Europe only, to limit the variation in political and social context, facilitating better generalization to the results to both the Norwegian and broader European context.

The studies from UK were rather old (1999, 2022, 2007, 2012) [18, 19, 23, 24], whereas the other were of more recent (2019–2022) [20–22, 25]. Knowledge and practice in the field may have developed over the last 20 years, but there was nothing that stood out as a difference between the results of the older and newer studies. Due to the lack of specific findings or conclusive evidence it was therefore difficult to make definitive statements about satisfaction with substance abuse treatment. Moreover, studies were carried out in both in-and out-patient settings, and each study had its own methodology and included questions. This further complicates drawing firm conclusion. Such variety, however, is part of the nature of scoping reviews. We included only scientific papers, and only papers published in English. By this, we may have lost relevant studies, but we assume that most relevant literature is published and available in English language. We did not perform a quality assessment of each paper, due to the broad nature of a scoping review and the varied nature of included studies.

### Implications

Our rationale for carrying out this scoping review was to identify areas of special importance to patient satisfaction among immigrant patients in mental health care, to ensure that our measurement tools are relevant and covers important aspects of care, also for immigrants. Our review reveal that aspects related to cultural appropriateness of services, language barriers and perceived respect for patients` background are important for satisfaction with services among immigrants, and that these aspects should be included also in quantitative measurements of patient experience and satisfaction in the future. Research is needed on how these aspects best can be included in future surveys. Furthermore, these areas will be of importance in quality development of mental health services in an increasingly heterogenous Europe. In the literature search, no studies regarding patient experiences among immigrant in substance abuse treatment were found, except one among men with co-occurring substance abuse- and mental health disorders. This calls for research on how such health care services are experienced by patients with immigrant background.

## Conclusion

Our scoping review highlights a need to raise awareness about the importance of respect and understanding for patients` cultural background and communication needs in improving mental health treatment given to patients with an immigrant or ethnic minority background. For immigrants` needs to be included in service quality improvement, these aspects should also be taken into quantitative and routine measures of patient experiences.

### Supplementary Information

**Additional file 1: Appendix 1.** Preferred Reporting Items for Systematic reviews and Meta-Analyses extension for Scoping Reviews (PRISMA-ScR) Checklist.

**Additional file 2: Appendix 2.** Search strategy.

**Additional file 3: Appendix 3.** Supplementary Table 1. List of articles excluded in full-text reading. Supplementary Table 2. Details of included studies.

**Additional file 4: Appendix 4.** Data extraction form, modified from Peters et al, 2020*.

## Data Availability

The scoping review is based on articles available in the databases searched.

## References

[1] Union OE (2018). Health at a Glance: Europe 2018. State of Health in the EU Cycle.

[2] Close C, Kouvonen A, Bosqui T, Patel K, O'Reilly D, Donnelly M (2016). The mental health and wellbeing of first generation migrants: a systematic-narrative review of reviews. Global Health.

[3] Abebe DS, Lien L, Hjelde KH (2014). What we know and don't know about mental health problems among immigrants in Norway. J Immigr Minor Health.

[4] Gilliver SC, Sundquist J, Li X, Sundquist K (2014). Recent research on the mental health of immigrants to Sweden: a literature review. Eur J Pub Health.

[5] Norredam M, Garcia-Lopez A, Keiding N, Krasnik A (2009). Risk of mental disorders in refugees and native Danes: a register-based retrospective cohort study. Soc Psychiatry Psychiatr Epidemiol.

[6] Berg JE (2009). The level of non-Western immigrants' use of acute psychiatric care compared with ethnic Norwegians over an 8-year period. Nord J Psychiatry.

[7] Abebe DS, Lien L, Elstad JI (2017). Immigrants' utilization of specialist mental healthcare according to age, country of origin, and migration history: a nation-wide register study in Norway. Soc Psychiatry Psychiatr Epidemiol.

[8] Doyle C, Lennox L, Bell D (2013). A systematic review of evidence on the links between patient experience and clinical safety and effectiveness. BMJ Open.

[9] Fernandes S, Fond G, Zendjidjian XY, Baumstarck K, Lançon C, Berna F (2020). Measuring the patient experience of mental health care: a systematic and critical review of patient-reported experience measures. Patient Prefer Adherence.

[10] Haugum M, Iversen HH, Helgeland J, Lindahl AK, Bjertnaes O (2019). Patient experiences with interdisciplinary treatment for substance dependence: an assessment of quality indicators based on two national surveys in Norway. Patient Prefer Adherence.

[11] Woodward S, Berry K, Bucci S (2017). A systematic review of factors associated with service user satisfaction with psychiatric inpatient services. J Psychiatr Res.

[12] Iversen HH, Haugum M, Bjertnaes O (2022). Reliability and validity of the Psychiatric Inpatient Patient Experience Questionnaire - Continuous Electronic Measurement (PIPEQ-CEM). BMC Health Serv Res.

[13] Munn Z, Pollock D, Khalil H, Alexander L, McLnerney P, Godfrey CM (2022). What are scoping reviews? Providing a formal definition of scoping reviews as a type of evidence synthesis. JBI Evid Synth.

[14] Peters MD, Godfrey C, McInerney P, Munn Z, Tricco AC, Khalil H. Scoping reviews. In: Aromatis E MZ, editor. JBI Manual for Evidence Synthesis: Joanna Briggs Institute; 2020. 1–24.

[15] Tricco AC, Lillie E, Zarin W, O'Brien KK, Colquhoun H, Levac D (2018). PRISMA extension for scoping reviews (PRISMA-ScR): checklist and explanation. Ann Intern Med.

[16] Danielsen KGA (2007). Kornør H Måling av brukererfaringer med avhengighetsbehandling: En litteraturgjennomgang av validerte måleinstrumenter Oslo.

[17] Crow R, Gage H, Hampson S, Hart J, Kimber A, Storey L, Thomas H (2002). The measurement of satisfaction with healthcare: implications for practice from a systematic review of the literature. Health Technol Assess.

[18] Greenwood N, Key A, Burns T, Bristow M, Sedgwick P (1999). Satisfaction with in-patient psychiatric services. Relationship to patient and treatment factors. Br J Psychiatry.

[19] Boydell J, Morgan C, Dutta R, Jones B, Alemseged F, Dazzan P (2012). Satisfaction with inpatient treatment for first-episode psychosis among different ethnic groups: a report from the UK ÆSOP study. Int J Soc Psychiatry.

[20] Lindberg LG, Mundy SS, Kristiansen M, Johansen KS, Carlsson J (2019). Satisfaction with mental health treatment among patients with a non-Western migrant background: a survey in a Danish specialized outpatient mental health clinic. Eur J Pub Health.

[21] Anderson K, Giacco D, Bird V, Bauer M, Pfennig A, Lasalvia A (2021). Do outcomes of psychiatric hospital treatment differ for migrants and non-migrants?. Soc Psychiatry Psychiatr Epidemiol.

[22] Gaigl G, Täumer E, Allgöwer A, Becker T, Breilmann J, Falkai P (2022). The role of migration in mental healthcare: treatment satisfaction and utilization. BMC Psychiatry.

[23] Bowl R (2007). The need for change in UK mental health services: South Asian service users’ views. Ethn Health.

[24] Bhui K, Chandran M, Sathyamoorthy G (2002). Mental health assessment and south Asian men. Int Rev Psychiatry.

[25] Kour P, Lien L, Kumar B, Biong S, Pettersen H (2020). Treatment experiences with Norwegian health care among immigrant men living with co-occurring substance use-and mental health disorders. Subst Abuse.

[26] Mangrio E, Sjögren FK (2017). Refugees' experiences of healthcare in the host country: a scoping review. BMC Health Serv Res.

[27] Detollenaere J, Hanssens L, Schäfer W, Willems S (2018). Can you recommend me a good GP? Describing social differences in patient satisfaction within 31 countries. Int J Qual Health Care.

[28] Goetz K, Bungartz J, Szecsenyi J, Steinhaeuser J (2015). How do patients with a Turkish background evaluate their medical care in Germany? An observational study in primary care. Patient Prefer Adherence.

[29] Wiking E, Saleh-Stattin N, Johansson SE, Sundquist J (2009). Immigrant patients' experiences and reflections pertaining to the consultation: a study on patients from Chile, Iran and Turkey in primary health care in Stockholm Sweden. Scand J Caring Sci.

[30] Ogden J, Jain A (2005). Patients' experiences and expectations of general practice: a questionnaire study of differences by ethnic group. Br J Gen Pract.

