# Supplementary material for: Immigrant and ethnic minority patients` reported experiences in psychiatric care in Europe – a scoping review
Source: BMC Health Serv Res. 2023 Nov 21;23:1281. doi: 10.1186/s12913-023-10312-1 (PMC10664498; doi:10.1186/s12913-023-10312-1)
Supplement: Supplementary file 2 — Additional file 2: Appendix 2. Search strategy. [file 12913_2023_10312_MOESM2_ESM.docx]

## Search strategy

Total hits: 2107

After removing duplicates: 1253

| **Aim** | | | | |
| --- | --- | --- | --- | --- |
| Experiences among former or current adult immigrant inpatients in psychiatric care or substance abuse treatement | | | | |
| **Spørsmålet i PICO-format** | | | | |
| **Population**  **(pasient)** | **Intervention**  **(tiltak)** | **Comparison**  **(samanlikning)** | **Outcome**  **(utfall)** |  |
| Immigrants | Inpatient psychiatric care or Inpatient substance abuse treatement |  | Patient experiences |  |

**Database:** Ovid MEDLINE(R) and Epub Ahead of Print, In-Process, In-Data-Review & Other Non-Indexed Citations, Daily and Versions <1946 to December 14, 2022>

**Date:** 15.12.22

**Number of hits:** 493

| 1 | Residential Treatment/ | 3307 |
| --- | --- | --- |
| 2 | exp Mental Disorders/ or exp Substance-Related Disorders/ | 1431405 |
| 3 | 1 and 2 | 2535 |
| 4 | Psychiatric department, Hospital/ or Hospitals, Psychiatric/ or Emergency Services, Psychiatric/ or Therapeutic Community/ | 36184 |
| 5 | ("speciali#ed mental health service?" or therapeutic communit* or ((mental or psychiatric) adj3 ("inpatient clinic?" or ward? or department? or unit? or hospital* or institution? or primary health care or primary care or community care or residential care or residential treatment?)) or asylum or (emergency adj1 psychiatric adj1 service?)).tw,kf. | 43881 |
| 6 | Substance Abuse Treatment Centers/ | 5425 |
| 7 | ((drug or substance or addiction or dependence or alcohol* or opioid? or opiate) adj3 ("inpatient clinic?" or center? or centre? or ward? or unit? or department? or rehab* or treatment? or therap* or intervention? or primary health care or primary care or community care or residential care)).tw,kf. | 224670 |
| 8 | or/3-7 | 289674 |
| 9 | Patient Satisfaction/ or Patient Reported Outcome Measures/ | 100043 |
| 10 | (((consumer? or patient? or user? or client? or care or healthcare or service or treatment) adj3 (experience* or satisfaction or assessment* or evaluat* or rating* or opinion* or judg* or perception* or perceive? or perspective* or "point-of-view")) or (("patient reported" or "self reported" or patientreported or selfreported) adj (outcome* or satisfaction or experience* or perception? or preference*)) or PROM or PROMs or PREM or PREMs).tw,kf. | 738697 |
| 11 | 9 or 10 | 783666 |
| 12 | exp "Emigrants and Immigrants"/ or Refugees/ or Minority Groups/ or "Emigration and Immigration"/ or Undocumented Immigrants/ or Minority Health/ | 67515 |
| 13 | (immigrant? or emigrant? or migrant? or emigration? or immigration? or emigrate? or immigrate? or foreigner? or alien? or refugee? or "displaced people" or resettle* or "race/ethnic*" or "racial/ethnic*" or race or racial or ethnic* or "geographic origin?" or multi cultural* or multicultural* or "newly arrived" or ((family or families) adj2 reuni*) or minorit* or (asylum adj1 seek*) or (border adj1 crossing?) or ((residential or geographical) adj mobility)).tw,kf. | 426616 |
| 14 | 12 or 13 | 446609 |
| 15 | 8 and 11 and 14 | 793 |
| 16 | limit 15 to yr="2012 -Current" | 493 |

**Database:** Embase <1974 to 2022 December 14>

**Date:** 15.12.22

**Number of hits:** 416

| 1 | residential care/ | 13053 |
| --- | --- | --- |
| 2 | exp mental disease/ or substance abuse/ or alcohol abuse/ | 2543639 |
| 3 | 1 and 2 | 5540 |
| 4 | psychiatric department/ or therapeutic community/ | 11160 |
| 5 | ("speciali#ed mental health service?" or therapeutic communit* or ((mental or psychiatric) adj3 ("inpatient clinic?" or ward? or department? or unit? or hospital* or institution? or primary health care or primary care or community care or residential care or residential treatment?)) or asylum or (emergency adj1 psychiatric adj1 service?)).tw,kf. | 53321 |
| 6 | drug dependence treatment/ or alcohol rehabilitation program/ | 9409 |
| 7 | ((drug or substance or addiction or dependence or alcohol* or opioid? or opiate) adj3 ("inpatient clinic?" or center? or centre? or ward? or unit? or department? or rehab* or treatment? or therap* or intervention? or primary health care or primary care or community care or residential care)).tw,kf. | 306263 |
| 8 | or/3-7 | 368534 |
| 9 | patient satisfaction/ or patient-reported outcome/ | 203469 |
| 10 | (((consumer? or patient? or user? or client? or care or healthcare or service or treatment) adj3 (experience* or satisfaction or assessment* or evaluat* or rating* or opinion* or judg* or perception* or perceive? or perspective* or "point-of-view")) or (("patient reported" or "self reported" or patientreported or selfreported) adj (outcome* or satisfaction or experience* or perception? or preference*)) or PROM or PROMs or PREM or PREMs).tw,kf. | 1145061 |
| 11 | 9 or 10 | 1227700 |
| 12 | exp migrant/ or refugee/ or minority group/ or exp migration/ or undocumented immigrant/ or minority health/ | 105954 |
| 13 | (immigrant? or emigrant? or migrant? or emigration? or immigration? or emigrate? or immigrate? or foreigner? or alien? or refugee? or "displaced people" or resettle* or "race/ethnic*" or "racial/ethnic*" or race or racial or ethnic* or "geographic origin?" or multi cultural* or multicultural* or "newly arrived" or ((family or families) adj2 reuni*) or minorit* or (asylum adj1 seek*) or (border adj1 crossing?) or ((residential or geographical) adj mobility)).tw,kf. | 584836 |
| 14 | 12 or 13 | 619694 |
| 15 | 8 and 11 and 14 | 1256 |
| 16 | limit 15 to "remove medline records" | 540 |
| 17 | limit 16 to yr="2012 -Current" | 416 |

**Database:** APA PsycInfo <1806 to December Week 1 2022>

**Date:** 15.12.22

**Number of hits:** 354

| 1 | Psychiatric units/ or Psychiatric Hospitals/ or Psychiatric hospitalization/ or Therapeutic Community/ | 20020 |
| --- | --- | --- |
| 2 | ("speciali#ed mental health service?" or therapeutic communit* or ((mental or psychiatric) adj3 ("inpatient clinic?" or ward? or department? or unit? or hospital* or institution? or primary health care or primary care or community care or residential care or residential treatment?)) or asylum or (emergency adj1 psychiatric adj1 service?)).tw. | 47792 |
| 3 | Drug Rehabilitation/ or Alcohol Rehabilitation/ | 30279 |
| 4 | ((drug or substance or addiction or dependence or alcohol* or opioid? or opiate) adj3 ("inpatient clinic?" or center? or centre? or ward? or unit? or department? or rehab* or treatment? or therap* or intervention? or primary health care or primary care or community care or residential care)).tw. | 82517 |
| 5 | or/1-4 | 142596 |
| 6 | Client Satisfaction/ or Patient Reported Outcome Measures/ | 6840 |
| 7 | (((consumer? or patient? or user? or client? or care or healthcare or service or treatment) adj3 (experience* or satisfaction or assessment* or evaluat* or rating* or opinion* or judg* or perception* or perceive? or perspective* or "point-of-view")) or (("patient reported" or "self reported" or patientreported or selfreported) adj (outcome* or satisfaction or experience* or perception? or preference*)) or PROM or PROMs or PREM or PREMs).tw. | 182500 |
| 8 | 6 or 7 | 183395 |
| 9 | "Racial and Ethnic Groups"/ or exp Human migration/ or Minority Groups/ or Immigration/ or Asylum Seeking/ | 68646 |
| 10 | (immigrant? or emigrant? or migrant? or emigration? or immigration? or emigrate? or immigrate? or foreigner? or alien? or refugee? or "displaced people" or resettle* or "race/ethnic*" or "racial/ethnic*" or race or racial or ethnic* or "geographic origin?" or multi cultural* or multicultural* or "newly arrived" or ((family or families) adj2 reuni*) or minorit* or (asylum adj1 seek*) or (border adj1 crossing?) or ((residential or geographical) adj mobility)).tw. | 270026 |
| 11 | 9 or 10 | 276929 |
| 12 | 5 and 8 and 11 | 736 |
| 13 | limit 12 to yr="2012 -Current" | 354 |

**Database:** Cochrane Database of Systematic Reviews

Issue 12 of 12, December 2022

Cochrane Central Register of Controlled Trials

Issue 11 of 12, November 2022

**Date:** 15.12.22

**Number of hits:** 103 (3 reviews, 100 trials)

| #1 | [mh ^"Residential Treatment"] | 180 |
| --- | --- | --- |
| #2 | [mh "Mental Disorders"] | 83241 |
| #3 | [mh "Substance-Related Disorders"] | 16645 |
| #4 | #2 or #3 | 83913 |
| #5 | #1 and #4 | 166 |
| #6 | [mh ^"Psychiatric department, Hospital"] | 102 |
| #7 | [mh ^"Hospitals, Psychiatric"] | 253 |
| #8 | [mh ^"Emergency Services, Psychiatric"] | 50 |
| #9 | [mh ^"Therapeutic Community"] | 53 |
| #10 | ((("specialized mental health" or "specialised mental health") NEXT service?) or (therapeutic NEXT communit*) or ((mental or psychiatric) NEAR/3 ("inpatient clinic" or "inpatient clinics" or ward? or department? or unit? or hospital* or institution? or "primary health care" or "primary care" or "community care" or "residential care" or "residential treatment" or "residential treatments")) or asylum or (("emergency psychiatric" or "psychiatric emergency") NEAR/1 service?)):ti,ab | 3022 |
| #11 | [mh ^"Substance Abuse Treatment Centers"] | 362 |
| #12 | ((drug or substance or addiction or dependence or alcohol* or opioid? or opiate) NEAR/3 ("inpatient clinic" or "inpatient clinics" or center? or centre? or ward? or unit? or department? or rehab* or treatment? or therap* or intervention? or "primary health care" or "primary care" or "community care" or "residential care")):ti,ab | 39921 |
| #13 | {or #5-#12} | 43087 |
| #14 | [mh ^"Patient Satisfaction"] | 12105 |
| #15 | [mh ^"Patient Reported Outcome Measures"] | 1008 |
| #16 | (((consumer? or patient? or user? or client? or care or healthcare or service or treatment) NEAR/3 (experience* or satisfaction or assessment* or evaluat* or rating* or opinion* or judg* or perception* or perceive? or perspective* or "point-of-view")) or (("patient reported" or "self reported" or patientreported or selfreported) NEXT (outcome* or satisfaction or experience* or perception? or preference*)) or PROM or PROMs or PREM or PREMs):ti,ab | 144169 |
| #17 | {or #14-#16} | 150137 |
| #18 | [mh "Emigrants and Immigrants"] | 204 |
| #19 | [mh ^"Refugees"] | 173 |
| #20 | [mh ^"Minority Groups"] | 414 |
| #21 | [mh ^"Emigration and Immigration"] | 67 |
| #22 | [mh ^"Undocumented Immigrants"] | 1 |
| #23 | [mh ^"Minority Health"] | 26 |
| #24 | (immigrant? or emigrant? or migrant? or emigration? or immigration? or emigrate? or immigrate? or foreigner? or alien? or refugee? or "displaced people" or resettle* or "race/ethnic*" or "racial/ethnic*" or race or racial or ethnic* or (geographic NEXT origin?) or (multi NEXT cultural*) or multicultural* or "newly arrived" or ((family or families) NEAR/2 reuni*) or minorit* or (asylum NEAR/1 seek*) or (border NEAR/1 crossing?) or ((residential or geographical) NEXT mobility)):ti,ab | 25609 |
| #25 | {or #18-#24} | 25704 |
| #26 | #13 and #17 and #25 | 148 |
| #27 | #26 with Cochrane Library publication date Between Jan 2012 and Dec 2022, in Cochrane Reviews | 3 |
| #28 | #26 with Publication Year from 2012 to 2022, in Trials | 100 |

**Database:** Cinahl via Ebsco

**Date:** 15.12.22

**Number of hits:** 120

| S1 | (MH "Hospitals, Psychiatric") or (MH "Psychiatric units") or (MH "Community Mental Health Nursing") | 11,210 |
| --- | --- | --- |
| S2 | TI (((specialized or specialised) W0 "mental health service#) or "therapeutic communit*" or ((mental or psychiatric) N2 ("inpatient clinic#" or ward# or department# or unit# or hospital* or institution# or "primary health care" or "primary care" or "community care" or "residential care" or "residential treatment#")) or asylum or (emergency N0 psychiatric N0 service#)) OR AB ("inpatient clinic#" or ((specialized or specialised) W0 "mental health service#) or "therapeutic communit*" or ((mental or psychiatric) N2 (ward# or department# or unit# or hospital* or institution# or "primary health care" or "primary care" or "community care" or "residential care" or "residential treatment#")) or asylum or (emergency N0 psychiatric N0 service#)) | 6,415 |
| S3 | (MH "Substance use rehabilitation program+") | 0 |
| S4 | TI ((drug or substance or addiction or dependence or alcohol* or opioid# or opiate) N2 ("inpatient clinic#" or center# or centre# or ward# or unit# or department# or rehab* or treatment# or therap* or intervention# or "primary health care" or "primary care" or "community care" or "residential care")) OR AB ((drug or substance or addiction or dependence or alcohol* or opioid# or opiate) N2 ("inpatient clinic#" or center# or centre# or ward# or unit# or department# or rehab* or treatment# or therap* or intervention# or "primary health care" or "primary care" or "community care" or "residential care")) | 64,213 |
| S5 | S1 OR S2 OR S3 OR S4 | 79,355 |
| S6 | (MH "Patient Satisfaction") or (MH "Consumer Satisfaction") or (MH "Patient-Reported Outcomes") | 77,017 |
| S7 | TI (((consumer# or patient# or user# or client# or care or healthcare or service or treatment) N2 (experience* or satisfaction or assessment* or evaluat* or rating* or opinion* or judg* or perception* or perceive# or perspective* or "point-of-view")) or (("patient reported" or "self reported" or patientreported or selfreported) W0 (outcome* or satisfaction or experience* or perception# or preference*)) or PROM or PROMs or PREM or PREMs) OR AB (((consumer# or patient# or user# or client# or care or healthcare or service or treatment) N2 (experience* or satisfaction or assessment* or evaluat* or rating* or opinion* or judg* or perception* or perceive# or perspective* or "point-of-view")) or (("patient reported" or "self reported" or patientreported or selfreported) W0 (outcome* or satisfaction or experience* or perception# or preference*)) or PROM or PROMs or PREM or PREMs) | 293,651 |
| S8 | S6 OR S7 | 338,352 |
| S9 | (MH "Refugees") or (MH "Minority Groups") or (MH "Immigrants+") or (MH "Residential Mobility+") | 50,565 |
| S10 | TI (immigrant# or emigrant# or migrant# or emigration# or immigration# or emigrate# or immigrate# or foreigner# or alien# or refugee# or "displaced people" or resettle* or "race/ethnic*" or "racial/ethnic*" or race or racial or ethnic* or "geographic origin#" or (multi W0 cultural*) or multicultural* or "newly arrived" or ((family or families) N1 reuni*) or minorit* or (asylum N0 seek*) or (border N0 crossing#) or ((residential or geographical) W0 mobility)) OR AB (immigrant# or emigrant# or migrant# or emigration# or immigration# or emigrate# or immigrate# or foreigner# or alien# or refugee# or "displaced people" or resettle* or "race/ethnic*" or "racial/ethnic*" or race or racial or ethnic* or "geographic origin#" or (multi W0 cultural*) or multicultural* or "newly arrived" or ((family or families) N1 reuni*) or minorit* or (asylum N0 seek*) or (border N0 crossing#) or ((residential or geographical) W0 mobility)) | 174,781 |
| S11 | S9 OR S10 | 191,294 |
| S12 | S5 AND S8 AND S11 | 362 |
| S13 | S12  Limiters - Published Date: 20120101-20221231 | 243 |
| S14 | S13  Limiters - Exclude MEDLINE records | 120 |

**Database:** Web of Science Core Collection

- WOS.SCI: 1987 to 2022

- WOS.AHCI: 1987 to 2022

- WOS.ESCI: 2017 to 2022

- WOS.SSCI: 1987 to 2022

**Date:** 15.12.22

**Number of hits:** 621

| 1 | TS=("speciali#ed mental health service$" or "therapeutic communit*" or ((mental or psychiatric) NEAR/2 ("inpatient clinic$" or ward$ or department$ or unit$ or hospital* or institution$ or "primary health care" or "primary care" or "community care" or "residential care" or "residential treatment$")) or asylum or (emergency NEAR/0 psychiatric NEAR/0 service$)) | \|Exact search | 41140 |
| --- | --- | --- | --- |
| 2 | TS=((drug or substance or addiction or dependence or alcohol* or opioid$ or opiate) NEAR/2 ("inpatient clinic$" or center$ or centre$ or ward$ or unit$ or department$ or rehab* or treatment$ or therap* or intervention$ or "primary health care" or "primary care" or "community care" or "residential care")) | \|Exact search | 203698 |
| 3 | #1 OR #2 | \|Exact search | 243143 |
| 4 | TS=(((consumer$ or patient$ or user$ or client$ or care or healthcare or service or treatment) NEAR/2 (experience* or satisfaction or assessment* or evaluat* or rating* or opinion* or judg* or perception* or perceive$ or perspective* or "point-of-view")) or (("patient reported" or "self reported" or patientreported or selfreported) NEAR/0 (outcome* or satisfaction or experience* or perception$ or preference*)) or PROM or PROMs or PREM or PREMs) | \|Exact search | 743376 |
| 5 | TS=("immigrant$" or "emigrant$" or "migrant$" or "emigration$" or "immigration$" or "emigrate$" or "immigrate$" or "foreigner$" or "alien$" or "refugee$" or "displaced people" or "resettle*" or "race/ethnic*" or "racial/ethnic*" or "race" or "racial" or "ethnic*" or "geographic origin$" or ("multi" NEAR/0 "cultural*") or "multicultural*" or "newly arrived" or (("family" or "families") NEAR/1 "reuni*") or "minorit*" or ("asylum" NEAR/0 "seek*") or ("border" NEAR/0 "crossing$") or (("residential" or "geographical") NEAR/0 "mobility")) | \|Exact search | 797242 |
| 6 | #3 AND #4 AND #5 | \|Exact search | 914 |
| 7 | #6  Timespan: 2012-01-01 to 2022-12-06 | \|Exact search | 621 |

|  |
| --- |
