# Supplementary material for: Immigrant and ethnic minority patients` reported experiences in psychiatric care in Europe – a scoping review
Source: BMC Health Serv Res. 2023 Nov 21;23:1281. doi: 10.1186/s12913-023-10312-1 (PMC10664498; doi:10.1186/s12913-023-10312-1)
Supplement: Supplementary file 3 — Additional file 3: Appendix 3. Supplementary Table 1. List of articles excluded in full-text reading. Supplementary Table 2. Details of included studies. [file 12913_2023_10312_MOESM3_ESM.docx]

Appendix 3

| **Supplementary table 1**. List of articles excluded in full-text reading | | |
| --- | --- | --- |
| **Authors (year)** | **Title** | **Reason for exclusion** |
| Zhen-Duan J. et al (2021) (9) | Ensuring access to high-quality substance use disorder treatment for Medicaid enrollees: A qualitative study of diverse stakeholders' perspectives | No explicit focus on immigrants/ethnic minorities |
| Aftab A. et al (2019) (10) | Impact of Psychiatric Hospitalization on Trust, Disclosure and Working Alliance with the Outpatient Psychiatric Provider: A Pilot Survey Study | Not reporting on patient experiences |
| Henderson RC. et al (2015) (11) | Mistrust of mental health services: ethnicity, hospital admission and unfair treatment | Not reporting on patient experiences |
|  |  | Not reporting on patient experiences |
| Achara-Abrahams I. (2012) (12) | Recovery management and African Americans: A report from the field | Reporting on a selected group (e.g forensic care or having additional diagnoses) |
| Huang MJ. et al (2020) (13) | Racial dynamics among clients in a residential substance use disorder treatment program in South Los Angeles and its impact on clients' treatment experiences | Reporting on a selected group (e.g forensic care or having additional diagnoses) |
| Keefe K. et al (2020) (14) | Understanding barriers and facilitators to therapeutic relationships in state psychiatric hospitals | Reporting on a selected group (e.g forensic care or having additional diagnoses) |
| Paull H. (2021) (15) | Consumer-based program evaluation focusing on client satisfaction at positive impact health centers' Decatur location | Reporting on a selected group (e.g forensic care or having additional diagnoses) |
| Mays VM. et al (2017) (16) | Perceived Discrimination in Health Care and Mental Health/Substance Abuse Treatment Among Blacks, Latinos, and Whites | Survey carried out in a general population, not a user-group |
| Rabiee F. et al ( 2013) (17) | Being understood, being respected: An evaluation of mental health service provision from service providers and users' perspectives in Birmingham, UK | Survey carried out in a general population, not a user-group |
| Vincent F. et al (2013) (18) | Asylum-seekers' experiences of trauma-focused cognitive behaviour therapy for post-traumatic stress disorder: a qualitative study | Reporting on one single treatment method |
| Jones LV. et al (2015) (19) | A qualitative study of black women's experiences in drug abuse and mental health services | Not from Europe |
| Colucci E. et al (2017) (20) | Improving access to and engagement with mental health services among young people from refugee backgrounds: Service user and provider perspectives | Not from Europe |
| Hargons CN. et al (2022) (21) | ?Can we get a Black rehabilitation center?? Factors impacting the treatment experiences of Black people who use opioids | Not from Europe |
| Majumder P. et al (2015) (22) | This doctor, I not trust him, I'm not safe': the perceptions of mental health and services by unaccompanied refugee adolescents | Children and adolescents` mental health care |
| Majumder P. et al (2019) (23) | Potential barriers in the therapeutic relationship in unaccompanied refugee minors in mental health | Children and adolescents` mental health care |
| Coelho H. et al (2022) (24) | Experiences of children and young people from ethnic minorities in accessing mental health care and support: rapid scoping review | Review on a related topic, screened for additional relevant articles |
| De Kock C. (2020) (25) | Cultural competence and derivatives in substance use treatment for migrants and ethnic minorities: what's the problem represented to be? | Review on a related topic, screened for additional relevant articles |
| Mangrio E. et al (2017) (26) | Refugees' experiences of healthcare in the host country: a scoping review | Review on a related topic, screened for additional relevant articles |
| Bansal N. et al (2022) (27) | Understanding ethnic inequalities in mental healthcare in the UK: A meta-ethnography | Review on a related topic, screened for additional relevant articles |

| **Supplementary table 2**. Details of included studies | | | | | | |
| --- | --- | --- | --- | --- | --- | --- |
| **Authors (year)** | **Context** | **Study design (study year)** | **Participants** | **Migration background**  **definition** | **Patient experiences assessed (questionnaire used if relevant)** | **Results of relevance to scoping review** |
| Anderson K. et al (2021) (1) | Belgium, Germany, Italy, Poland, UK  Routine inpatient mental health treatment, 57 hospitals | Quantitative, part of observational, longitudinal study (2014, 2015) | N=6298 non-migrants, N=985 migrants  ≥18 years  47.6% women  Diagnosed with psychotic disorder, affective disorder or anxiety/somatization disorder and hospitalized in a general adult psychiatric inpatient unit. | Migrants defined as persons born in another country than the one in which they are treated | Client Assessment of Treatment Scale (CAT)  One index on satisfaction based on satisfaction in included items:  1) Do you believe you are receiving the right treatment/care for you?  2) Does your therapist/case manager/keyworker understand you and is he/she engaged in your treatment?  3) Are relations with other staff members pleasant for you?  4) Do you believe you are receiving the right medication for you?  5) Do you believe the other elements of treatment/care here are right for you?  6) Do you feel respected and regarded well?  7) Has treatment/care here been helpful for you? | Migrants reported lower satisfaction with treatment than others, and also had a lower rate of suicide attempts. |
| Bhui K. et al (2002) (2) | UK  Out- and inpatient mental health services, including one adapted to South Asians | Qualitative individual in-depth semistructural interviews (2000) | N=8 men with south Asian background (India, Sri Lanka, Pakistan, Bangladesh)  19-62 years. | 1st and 2nd generation immigrants | Interviews about experiences in the mental health services | Main themes  (1) Clinical contact (Many expressed that it had been a support to have a relative with them)  (2) Professional role (Some, but not all, experienced that the staff and/or doctors did not explain their role or what was about to happen)  (3) Language and interpreters (Interpreter not offered, although it would have helped)  (4) Ethnicity and gender (Some, but not all, would have preferred a South Asian practitioner who could better understand their cultural and religious background, and opportunities to discuss their cultural background in general)  (5) Religion and culture (All users said it would have been useful to have the opportunity to discuss the role of their culture, but none had been given the opportunity)  (6) Understanding the problem (Some, but not all were satisfied with the level of explanations given about their diagnosis)  (7) Reflection on assessment (Both positive and negative aspects were mentioned)  (8) Treatment (Some were satisfied with the treatment, others felt they did not get enough information or that they were forced to take treatment. They were not offered interpreter although would have helped.) |
| Bowl R. (2007) (3) | UK  Local mental health care, inpatient and outpatient (some services specifically catering to South Asian mental health service users) | Qualitative individual and focus-group interviews (year not given) | N=26 users with Indian or Pakistani origin  15 women, 11 men  21- ≥60 years  Long term mental health care users | Pakistani or Indian origin | Experiences in mental health care were discussed, with a broad agenda about inviting participants to talk about  (1) their lives  (2) share their experiences of mental distress and of mental health services  (3) to identify how they would like to see support for themselves enhanced | Participants highlighted challenges related to communication and a deep understanding of what was communicated, expressing themselves in English language, lack of interpreters (although the need had been identified).  Tests (e.g. mental state) were insensitive, as users did not have the prerequisite skills and were scored poorly because of that.  Lack of cultural understanding among staff, and practical barriers to practice religion (e.g. place to pray  Processes of institutional exclusion reinforced these effects with South Asian service users feeling that it was unsafe to share their particular concerns within many service settings. They also felt that too little attention was paid to the particular lack of understanding within their families and communities of the nature and impact of mental illness and the effect that has on support and care available to them. |
| Boydell J et al (2012) (4) | UK  In-patients (acute psychiatric wards), three centres | Quantitative, cross-sectional questionnaire (1997-1999) | N=216  50.5% males  Mean age 31.4 years  50% White British, 27% Black Caribbean, 13% Black African, 5 % other ethnicity  Patients with first-episode psychosis. | Self-ascribed ethnicity | Acute Service Study Questionnaire  21 items of satisfaction, compiled to an overall satisfaction score:  1) I am not kept active enough during the day  2) I did not receive help quickly enough in the beginning  3) I do not see the doctor enough  4) I am receiving the right treatment  5) The ward is too noisy  6) I am satisfied with the admission process  7) In terms of treatment, I am satisfied with the drugs I have been given to take  8) I am satisfied with other treatment (non-drug) I am receiving  9) The behaviour of other patients is worrying  10) I can’t get enough privacy  11) I am not getting enough treatment for my problem  12) The ward has a pleasant atmosphere  13) Access to television is unsatisfactory  14) Personal belongings on the ward are safe from theft  15) The ward is too crowded  16) I would recommend treatment here to another relative or friend if needed  17) I do not feel safe on the ward  18) I get sufficient care from the nurses  19) I do not get bored here  20) The service was sensitive to my cultural/religious practices  21) Overall I feel better | Black patients were less likely to perceive they received the right treatment and less satisfied with medication than White, but equally satisfied with nursing and social care. Black African patients were less satisfied with non-pharmacological treatments than White patients. No ethnic differences in 16 of 21 individual domains of satisfaction. |
| Gaigl G. et al (2022) (5) | Germany  Mental health care, in-patient and day hospital. Ten departments included. | Quantitative, cross-sectional survey (2019) | N=387, of which N=72 with a migrant background (1^st^ or 2^nd^ generation)  56,8% women  18-65 years  Diagnosed with schizophrenia, bipolar disorder or depression and duration of psychiatric illness≥2 years | Assessed by self-identification: “Do you have a migration background?”. If yes, participants were asked: “Yes, I am a migrant myself.” (1st generation migrant) vs. “Yes, at least one of my parents is a migrant.” (2nd generation migrant). | VSSS-54:  Overall Satisfaction (three items: satisfaction with the amount of help received, the kind of treatment services, the overall treatment services)  Professionals’ Skills and Behavior (24 items: satisfaction with professionals’ behavior, e.g. interpersonal skills)  Information (three items: satisfaction with information on disorders, therapies and services), Access (two items: satisfaction with service location and costs)  Efficacy (eight items: satisfaction with overall and specific aspects of efficacy of service, e.g. social skills)  Relatives Involvement (six items: satisfaction with help given to relatives/ persons of trust)  Types of Intervention (17 items: satisfaction with and use of e.g. medical prescription, psychotherapy).  CAN-EU:  Basic, Functioning, Health, Social and Services.  The participants were asked whether there was a need regarding the individual domains in the past 4 weeks. In case of a need, the participant was asked whether adequate care was received | Patients with a migrant background higher Overall Satisfaction and satisfaction with Relatives Involvement than non-migrants.  1st generation migrants had higher Overall Satisfaction and satisfaction with Professionals Skills and Behaviour, Efficacy and Relatives Involvement compared to 2nd generation or non-migrant background.  No differences between both groups were identified in met and unmet treatment needs and use of supply services (psychiatric, psychotherapeutic, and psychosocial treatment |
| Greenwood N. et al (1999) (6) | UK  In-patient from two hospitals, six acute psychiatric wards | Quantitative with a standard questionnaire and qualitative semi-structured interviews with questionnaire with 113 questions regarding patient experiences | N=433  33% with other ethnicity than White | Two comparisonsWhite (N=294) vs non-White (N=90)  and  White (N=294) vs Black (N=53).  Ethnicity as defined by staff or in field notes. | Single question on satisfaction  Overall score on satisfaction based on a 8-item questionnaire (Client Satisfaction Experience Questionnaire (CSQ 8))  Semi structured interviews including 11 domains:  1) How patient came into treatment  2) Most and least helpful aspects of treatments  3) Treatment and its explanations  4) Ward activities  5) Food  6) Privacy  7) Staff interpersonal skills  8) Adverse events  9) Diagnoses  10) Ward rounds  11) Plans for discharge | No statistically significant relationships were found for ethnic groups regarding satisfaction (although a marginally higher proportion of White patients than either non-White or Black patients were satisfied).  More White than non-White patients reported adverse experiences, but the differences were not statistically significant.  White patients reported more adverse events than Black patients, the only statistical significance was for 'feeling afraid' |
| Kour P. et al (2020) (7) | Norway  Inpatient and outpatient substance abuse treatment and mental health services | Semi-structured, in-depth, qualitative interviews (2018-2019) | 10 men with substance abuse- and mental health disorder and immigrant background (1^st^ or 2^nd^ generation)  25-53 years | “Immigrants” defined as persons who were born or whose parents were born in low- and middle-income countries. In addition, we include as immigrants persons born abroad or in Norway of 2 foreign-born parents and 4 foreign-born grandparents | An interview guide, consisting of open-ended questions:  1) Can you please describe your experiences with treatment within Norwegian mental health and addiction services as a person with immigrant background?  2) Can you please tell us why according to you persons with immigrant background do not take the complete treatment?  3) Can you please describe your experiences with treatment when it comes to your needs as a person with immigrant background?  4) What kind of treatment do you wish for as a person with immigrant background in Norway? | Participants described their treatment experiences in mental health and addiction services in Norway in several areas:  (1) Lack of connection; a lack of connection between themselves and health professionals, lack of interest, not being listened to and unsatisfactory communication. The lack of connection was stronger when the HP only talked about their diagnosis and said nothing about contexts that were important for them, like their process of migration, living as an immigrant in Norway, reasons why they started using substances and discrimination in treatment settings.  (2) Lack of individually tailored treatment; The participants described experiencing a conventional and standard approach to treatment with no cultural sensitivity. Conventional group therapy where the participants felt mismatched in the group. Not being understood specifically in relation to their cultural beliefs, values and language.  (3) Stigma and discrimination; Participants faced discrimination in their communities due to their disorders, preventing access to treatment, but also discrimination within health services preventing getting the right help.  (4) Health professionals with multi-cultural competence; Having health professionals who had expertise in understanding different cultural backgrounds was helpful in completing treatment. They further mentioned that health professionals who recognized the special needs of immigrants led to a higher level of satisfaction with treatment.  (5) Care during and after treatment; Participants highlighted the importance of being treated with respect, being seen beyond the addiction, and also of aftercare. A few participants said that conversations about their past related to migration and settling in Norway and understanding their needs in relation to their past in the treatment process had a positive impact on them.  (6) Raising awareness and reducing stigma; Less stigma in their communities could have made it easier to seek help. Several also mentioned that having a supportive network of family, peers and role models was mentioned as particularly helpful, both during and after treatment. |
| Lindberg LG. et al (2019) (8) | Denmark  Outpatient mental health clinic (Competence Centre for Transcultural Psychiatry, which is a public tertiary outpatient mental health clinic) | Quantitative, cross-sectional survey (4 studies, 2009-2017) | N=686 non-Western migrants  322 women (49.6%), 364 (51.3%) men  ≥18 years  Diagnosed with PTSD or depression (excluded if diagnosed with severe psychotic disorder, addiction to psychoactive substances or in need of somatic or psychiatric hospitalization ).  ≥18 years  Diagnosed with PTSD or depression (excluded if diagnosed with severe psychotic disorder, addiction to psychoactive substances or in need of somatic or psychiatric hospitalization). | Non-Western migrants; refugees or family reunified to a refugee (country of origin: Iraq, Lebanon, ex-Yoguslavia, Afghanistan, Iran and others) | 10 items directly related to treatment satisfaction. (Questionnaire developed from literature reviews, questionnaire from other services and clinical experience.)  1. Overall treatment satisfaction  Have you found the treatment at CTP worth your time and efforts?  2. Improvement in wellbeing  Have you felt any improvements in your health or situation during your course of  treatment at CTP?  3. Influence on treatment  Do you feel that you have had an influence on your course of treatment at CTP?  4. Cultural understanding  Do you feel that CTP has understood and respected your cultural background?  5. Satisfied with medical doctor contact  Have you generally been satisfied with your contact to the medical doctor?  6. Satisfied with psychologist contact  Have you generally been satisfied with your contact to the psychologist?  7. Respect from medical doctor  Do you feel that the medical doctor at CTP has shown respect and consideration towards  you as a person?  8. Respect from psychologist  Do you feel that the psychologist at CTP has shown respect and consideration towards  you as a person?  9. Feeling more hopeful  Do you feel more hopeful about your future and your situation?  10. Received information about illness and treatment  Have you received the information about your illness and your treatment that you needed? | Overall satisfaction with treatment was high, and also satisfaction with cultural understanding, influence on own treatment and with the professionals. Fewer experienced improvements in wellbeing.  An experience of improvement and that their cultural background was respected and understood was associated with higher overall satisfaction. |

**References**

1. Anderson K, Giacco D, Bird V, Bauer M, Pfennig A, Lasalvia A, et al. Do outcomes of psychiatric hospital treatment differ for migrants and non-migrants? Social psychiatry and psychiatric epidemiology. 2021;56(11):1957-65.

2. Bhui K, Chandran M, Sathyamoorthy G. Mental health assessment and south Asian men. International Review of Psychiatry. 2002;14(1):52-9.

3. Bowl R. The need for change in UK mental health services: South Asian service users’ views. Ethnicity and Health. 2007;12(1):1-19.

4. Boydell J, Morgan C, Dutta R, Jones B, Alemseged F, Dazzan P, et al. Satisfaction with inpatient treatment for first-episode psychosis among different ethnic groups: A report from the UK ÆSOP study. International Journal of Social Psychiatry. 2012;58(1):98-105.

5. Gaigl G, Täumer E, Allgöwer A, Becker T, Breilmann J, Falkai P, et al. The role of migration in mental healthcare: treatment satisfaction and utilization. BMC psychiatry. 2022;22(1):1-13.

6. Greenwood N, Key A, Burns T, Bristow M, Sedgwick P. Satisfaction with in-patient psychiatric services. Relationship to patient and treatment factors. Br J Psychiatry. 1999;174:159-63.

7. Kour P, Lien L, Kumar B, Biong S, Pettersen H. Treatment experiences with Norwegian health care among immigrant men living with co-occurring substance use-and mental health disorders. Substance Abuse: Research and Treatment. 2020;14:1178221820970929.

8. Lindberg LG, Mundy SS, Kristiansen M, Johansen KS, Carlsson J. Satisfaction with mental health treatment among patients with a non-Western migrant background: a survey in a Danish specialized outpatient mental health clinic. European Journal of Public Health. 2019;29(4):700-5.

9. Zhen-Duan J, Fukuda M, DeJonckheere M, Falgas-Bague I, Miyawaki S, Khazi P, et al. Ensuring access to high-quality substance use disorder treatment for Medicaid enrollees: A qualitative study of diverse stakeholders' perspectives. Journal of Substance Abuse Treatment. 2021;129:108511.

10. Aftab A, LaGrotta C, Zyzanski SJ, Mishra P, Mehdi SMA, Brown K, et al. Impact of Psychiatric Hospitalization on Trust, Disclosure and Working Alliance with the Outpatient Psychiatric Provider: A Pilot Survey Study. Cureus. 2019;11(4):e4515.

11. Henderson RC, Williams P, Gabbidon J, Farrelly S, Schauman O, Hatch S, et al. Mistrust of mental health services: ethnicity, hospital admission and unfair treatment. Epidemiol Psychiatr Sci. 2015;24(3):258-65.

12. Achara-Abrahams I, Evans AC, Ortiz J, Lopez Villegas D, O'Dell J, Ali O, et al. Recovery management and African Americans: A report from the field. Alcoholism Treatment Quarterly. 2012;30(3):263-92.

13. Huang MJ, Grella C, Urada D, Beavers R. Racial dynamics among clients in a residential substance use disorder treatment program in South Los Angeles and its impact on clients' treatment experiences. J Ethn Subst Abuse. 2020:1-22.

14. Keefe K, Cardemil EV, Thompson M. Understanding barriers and facilitators to therapeutic relationships in state psychiatric hospitals. J Clin Psychol. 2020;76(1):195-209.

15. Paull H. Consumer-based program evaluation focusing on client satisfaction at positive impact health centers' Decatur location. Dissertation Abstracts International: Section B: The Sciences and Engineering. 2021;82(3-B):No Pagination Specified.

16. Mays VM, Jones AL, Delany-Brumsey A, Coles C, Cochran SD. Perceived Discrimination in Health Care and Mental Health/Substance Abuse Treatment Among Blacks, Latinos, and Whites. Medical Care. 2017;55(2):173-81.

17. Rabiee F, Smith P. Being understood, being respected: An evaluation of mental health service provision from service providers and users' perspectives in Birmingham, UK. International Journal of Mental Health Promotion. 2013;15(3):162-77.

18. Vincent F, Jenkins H, Larkin M, Clohessy S. Asylum-seekers' experiences of trauma-focused cognitive behaviour therapy for post-traumatic stress disorder: a qualitative study. Behav. 2013;41(5):579-93.

19. Jones LV, Hopson L, Warner L, Hardiman ER, James T. A qualitative study of black women's experiences in drug abuse and mental health services. Affilia: Journal of Women & Social Work. 2015;30(1):68-82.

20. Colucci E, Valibhoy M, Szwarc J, Kaplan I, Minas H. Improving access to and engagement with mental health services among young people from refugee backgrounds: Service user and provider perspectives. International Journal of Culture and Mental Health. 2017;10(2):185-96.

21. Hargons CN, Miller-Roenigk BD, Malone NJ, Mizelle DL, Atkinson JD, Stevens-Watkins DJ. ?Can we get a Black rehabilitation center?? Factors impacting the treatment experiences of Black people who use opioids. Journal of Substance Abuse Treatment. 2022;142:10.

22. Majumder P, O'Reilly M, Karim K, Vostanis P. 'This doctor, I not trust him, I'm not safe': the perceptions of mental health and services by unaccompanied refugee adolescents. Int J Soc Psychiatry. 2015;61(2):129-36.

23. Majumder P, Vostanis P, Karim K, O'Reilly M. Potential barriers in the therapeutic relationship in unaccompanied refugee minors in mental health. J Ment Health. 2019;28(4):372-8.

24. Coelho H, Price A, Kiff F, Trigg L, Robinson S, Thompson Coon J, et al. Experiences of children and young people from ethnic minorities in accessing mental health care and support: rapid scoping review. 2022.

25. De Kock C. Cultural competence and derivatives in substance use treatment for migrants and ethnic minorities: what's the problem represented to be? Soc Theory Health. 2020;18(4):358-94.

26. Mangrio E, Forss KS. Refugees' experiences of healthcare in the host country: a scoping review. Bmc Health Services Research. 2017;17:16.

27. Bansal N, Karlsen S, Sashidharan SP, Cohen R, Chew-Graham CA, Malpass A. Understanding ethnic inequalities in mental healthcare in the UK: A meta-ethnography. PLoS Med. 2022;19(12):e1004139.
