# Supplementary material for: Immigrant and ethnic minority patients` reported experiences in psychiatric care in Europe – a scoping review
Source: BMC Health Serv Res. 2023 Nov 21;23:1281. doi: 10.1186/s12913-023-10312-1 (PMC10664498; doi:10.1186/s12913-023-10312-1)
Supplement: Supplementary file 4 — Additional file 4: Appendix 4. Data extraction form, modified from Peters et al, 2020*. [file 12913_2023_10312_MOESM4_ESM.docx]

| Appendix 4. Data extraction form, modified from Peters et al, 2020* | |
| --- | --- |
| **Scoping Review Details** |  |
| Author |  |
| Publication year |  |
| Title |  |
| Aim |  |
| Country |  |
| Context |  |
| Participants (Number, age, sex) |  |
| Migration background definition |  |
| Study design |  |
| **Details/Results** |  |
| Questionnaire applied (if relevant) |  |
| Domains of experiences assessed |  |
| Results, main points relevant to scoping review study aim |  |

* Peters MD, Godfrey C, McInerney P, Munn Z, Tricco AC, Khalil H. Scoping reviews. In: Aromatis E MZ, editor. JBI Manual for Evidence Synthesis: Joanna Briggs Institute; 2020. p. 1-24.
